# Rapid lymphatic efflux limits cerebrospinal fluid flow to the brain

**DOI:** 10.1007/s00401-018-1916-x

**Published:** 2018-10-10

**Authors:** Qiaoli Ma, Miriam Ries, Yann Decker, Andreas Müller, Chantal Riner, Arno Bücker, Klaus Fassbender, Michael Detmar, Steven T. Proulx

**Affiliations:** 1 Institute of Pharmaceutical Sciences, Swiss Federal Institute of Technology, ETH Zurich, Vladimir-Prelog-Weg 1-5/10, HCI H398, 8093 Zurich, Switzerland; 2 Department of Neurology, University of the Saarland, 66421 Homburg, Saar Germany; 3 Clinic for Diagnostic and Interventional Radiology, Saarland University Medical Center, 66421 Homburg, Saar Germany

**Keywords:** Cerebrospinal fluid, Lymphatic vessel, Paravascular space, Anesthesia

## Abstract

The relationships between cerebrospinal fluid (CSF) and brain interstitial fluid are still being elucidated. It has been proposed that CSF within the subarachnoid space will enter paravascular spaces along arteries to flush through the parenchyma of the brain. However, CSF also directly exits the subarachnoid space through the cribriform plate and other perineural routes to reach the lymphatic system. In this study, we aimed to elucidate the functional relationship between CSF efflux through lymphatics and the potential influx into the brain by assessment of the distribution of CSF-infused tracers in awake and anesthetized mice. Using near-infrared fluorescence imaging, we showed that tracers quickly exited the subarachnoid space by transport through the lymphatic system to the systemic circulation in awake mice, significantly limiting their spread to the paravascular spaces of the brain. Magnetic resonance imaging and fluorescence microscopy through the skull under anesthetized conditions indicated that tracers remained confined to paravascular spaces on the surface of the brain. Immediately after death, a substantial influx of tracers occurred along paravascular spaces extending into the brain parenchyma. We conclude that under normal conditions a rapid CSF turnover through lymphatics precludes significant bulk flow into the brain.

**Electronic supplementary material:**

The online version of this article (10.1007/s00401-018-1916-x) contains supplementary material, which is available to authorized users.

## Introduction

The anatomical pathways and physiological mechanisms for the production, circulation and absorption of cerebrospinal fluid (CSF) and brain interstitial fluid (ISF) are currently an area of intense research focus. Historically, ISF is considered to be produced at the blood–brain barrier and to drain out of the brain towards the CSF [1, 14, 24, 53]. Pathways for a bulk flow of ISF from the brain parenchyma were found to be along peri-(or para-) vascular spaces (PVS) to reach the subarachnoid space (SAS) [13, 24, 53, 57] or along white matter fiber tracts to reach the ventricles [13, 46, 48]. However, several reports were published that ran counter to this view and suggested that a flow of CSF could occur from the SAS into the brain [8, 11, 40, 45, 52]. Of special note, Rennels et al. proposed a rapid CSF microcirculation to the brain with paravascular influx around arteries and efflux along veins. Although this concept was not fully accepted at the time [27], this work was recently cited as supporting evidence for a proposed glymphatic system of flow by Iliff et al. [29]. In experimental studies, tracers injected into the cisterna magna in mice were found with 2-photon imaging through a cranial window to enter the PVS around arteries penetrating into the cortex. At later time points tracers were also present in the PVS around veins. It was proposed that an astrocyte-facilitated convective flow existed through the parenchyma with influx along arteries and efflux along veins. Interestingly, this system was found to be more active during sleeping or anesthetized conditions compared to awake conditions [54]. However, at this time, these studies remain highly controversial and several groups have challenged various aspects of the concept [1, 3, 5, 19, 21, 25, 26, 50, 51].

While it is traditionally understood that CSF is absorbed mostly through arachnoid projections into the venous blood, we have recently demonstrated, using in vivo dynamic fluorescence imaging, that bulk outflow of CSF occurs predominantly through the lymphatic system in mice [37]. The major CSF outflow pathways were found along cranial nerves (e.g., olfactory and optic) to reach lymphatic vessels outside the skull, consistent with many previous reports [10, 32, 33, 39]. We have also demonstrated that lymphatic transport of interstitial fluid from skin and the peritoneal cavity is vastly increased during awake conditions compared to anesthetized conditions [44]. Therefore, it is of interest to determine how CSF outflow differs between awake and anesthetized mice and how these potential differences could influence the spread of CSF to the PVS of the brain. Thus, the first aim of the current study was to assess how lymphatic outflow from the CSF to the systemic blood is altered during awake conditions compared to anesthetized conditions. We next aimed to determine whether the outflow of CSF was correlated with the spread of tracers from the CSF into the PVS of the brain surface and brain parenchyma. Finally, we attempted to confirm whether an influx of tracers through the arterial PVS could be demonstrated under in vivo anesthetized conditions using through-skull near-infrared (NIR) imaging or by magnetic resonance imaging (MRI).

## Materials and methods

### Mice

Female C57BL/6J-*Tyr*^*c*-*J*^ albino or C57BL/6J wild type mice (Jackson Laboratories, Bar Harbor, ME) and Prox1-GFP [12] and SMMHC-GFP [55] reporter mice on C57BL/6J backgrounds were kept under specific pathogen–free conditions and used for experimental studies at the age of 2–3 months. All mouse experiments were approved by Kantonales Veterinaramt Zurich (license numbers 185/13, 196/13 and 161/16) and by the Landesamt für Gesundheit und Verbraucherschutz, Saarbruecken, Germany (license number: 15/2017), and performed following the regulations of the Swiss Federal Welfare Act (TSchG) and the European legislation on the protection of animals (Directive 2010/63/EU).

### Studies of CSF outflow during awake or anesthesia conditions

At 20 min before intracerebroventricular (i.c.v.) infusion, 0.1 mg/kg buprenorphine was injected subcutaneously. Mice were anesthetized by intraperitoneal injection of 80 mg/kg ketamine and 0.2 mg/kg medetomidine or inhalation of 2% isoflurane and fixed in a stereotaxic frame (RWD, San Diego, CA). For the awake group, lateral ventricle tracer infusion (as described below) was performed under 2% isoflurane and the mice were allowed to recover (within 5–10 min after infusion) and were awake for 60 min before imaging. Mice were observed to be active and behaving normally. For the isoflurane group, tracer infusion was performed under 2% isoflurane and the mice were kept anesthetized under 2% isoflurane for 60 min on a heating pad (37 °C) before imaging. For the ket/med group, 80 mg/kg ketamine and 0.2 mg/kg medetomidine were injected intraperitoneally before tracer infusion and the mice were kept anesthetized for 60 min on a heating pad (37 °C) before imaging. About 2 min before imaging at the saphenous vein, mice from the awake and isoflurane groups were given 80 mg/kg ketamine and 0.2 mg/kg medetomidine intraperitoneally. Mice were first imaged for the blood signal at the saphenous vein, then overdosed by intraperitoneal injection of 400 mg/kg ketamine and 1 mg/kg medetomidine for post-mortem imaging.

### Infusion of tracers into the lateral ventricle

The skull was thinned with a dental drill (RWD) at a location 0.95 mm lateral and 0.22 mm caudal to the bregma. A 34 G steel needle was inserted into the right lateral ventricle 2.35 mm ventral to the skull surface. For the standard protocol, 2.5 µL of 200 μM P40D680 or P40D800 [43] tracer at the speed of 1 µL/min was then infused with a syringe pump (Stoelting, Wood Dale, IL). For determination of the outflow dynamics in response to different volume infusions, alternate protocols involving 1.0 µL of 500 μM P40D680 at 0.4 µL/min and 5.0 µL of 100 μM P40D680 at 2 µL/min were used. The needle was left in place for 2.5 min and then slowly removed while observing if any significant backflow occurred. After tracer infusion, the injection hole in the skull was filled with bone wax (Ethicon, Somerville, NJ) and the scalp was sutured, except when in vivo through-skull imaging was subsequently carried out. Animals were excluded if significant backflow occurred or ex vivo analysis of brain slices indicated that the injection of tracer into the ventricle was not successful. For experiments where ventricular infusion was followed by dynamic contrast-enhanced MRI imaging, an identical protocol was used with the following modifications: 2.5 µL of a Gadospin D solution at 25 mM gadolinium (nanoPET Pharma GmbH, Germany) was infused instead of P40D680, a 33 G steel needle was used in combination with a NanoJet syringe pump (Chemyx, Stafford, CT) and the needle was left in place after infusion for 5 min.

### Infusion of tracers into the cisterna magna

Mice were anesthetized by intraperitoneal injection of 80 mg/kg ketamine and 0.2 mg/kg medetomidine, fixed in a stereotaxic frame (Kopf, Tujunga, CA) and the body temperature was maintained at 37 °C using a heating pad. A surgical procedure to access the cisterna magna was performed [30]. After a small skin incision over the occipital bone/cervical spinal cord was made, the three covering muscle layers were carefully dissected under a stereomicroscope using fine forceps and scissors. A beveled glass capillary micropipette (Sutter instruments, Novato, CA, USA) with a diameter of < 60 μm was made using a Sutter P97 Pipette puller (Sutter instruments) and was positioned perpendicular to the ear bars and advanced to penetrate the dura until resistance was overcome, indicating entry into the cisterna magna as previously described [2]. Overall, 5 µL of a Gadospin D solution at 25 mM gadolinium (nanoPET Pharma GmbH) was infused at the speed of 1 µL/min with a NanoJet syringe pump (Chemyx). After the infusion, the micropipette was left in place for 10 min to avoid reflux. Following its withdrawal, the wound was closed.

### NIR imaging of CSF lymphatic transport to systemic blood

For noninvasive imaging of tracer signals in blood [37], fur above the saphenous vein region was removed with a razor and depilation cream before the i.c.v infusion. At the desired time point after i.c.v. infusion, mice were anesthetized with ket/met as described above. Mice were then positioned under a Zeiss StereoLumar.V12 stereomicroscope with AxioVision software (Carl Zeiss, Feldbach, Switzerland) and a Photometrics Evolve 512 camera (Photometrics, Tucson, AZ) in a supine position on a heating pad (37 °C) for imaging. The autofluorescence signal on the GFP channel was used to position the saphenous blood vessels at 64 × zoom. An image under the Cy5 filter was acquired with exposure time and camera gain settings of 200 ms and 200, respectively.

For dynamic imaging of tracer outflow to blood with different infusion rates, mice were positioned within 5 min after the completion of infusion under the stereomicroscope as above. Dynamic imaging was initiated 5 min after the completion of the ventricle infusion by acquisition of a sequence of images (1 image every 15 s for 55 min) with a Cy5 filter set to monitor the NIR signal of the saphenous vein. Exposure time and camera gain settings were 200 ms and 200, respectively.

### Assessment of lymphatic transport to blood

Using AxioVision software, a circular region of interest (ROI) of radius 100 μm was placed over the saphenous vein on the acquired images. The mean signal intensity was then recorded within this region. For quantification of signal enhancement, tissue background signals were subtracted using the mean values from three uninjected mice with the same image acquisition settings.

For the dynamic imaging, a table of fluorescence intensity in counts versus time was exported into Microsoft Excel using the measure profile function. Since there was a slight loss of signal at the beginning of the scans due to photo bleaching of tissue autofluorescence, baseline intensity in counts was calculated as an average signal of the lowest ten consecutive imaging frames. This baseline intensity was then subtracted from the fluorescence intensity values in order to plot fluorescent signal enhancement versus time in min. The transport time to blood was determined as the point at which signal enhancement value was 100 counts above baseline levels.

### Analysis of tracer distribution on the brain surface and in CNS-draining lymph nodes

Images of P40D680 tracer spread on the surfaces of the brain and within the deep cervical and mandibular lymph nodes were acquired with a Zeiss AxioZoom V16 microscope and a QImaging OptiMOS sCMOS camera (QImaging, Surrey, Canada) combined with a light-emitting diode illumination system pE-4000 (CoolLED Ltd, Andover, UK) and ZEN 2 software (Carl Zeiss, Feldbach, Switzerland). In excised brains, images were acquired over the contralateral dorsal hemisphere (20 × , 20 ms exposure) and of the entire ventral side of the brain (11.2 × , 200 ms exposure). Images of lymph nodes were acquired in situ at 25 × and 200 ms exposure time. Since there were no apparent differences in signal in the lymph nodes on the injected and contralateral sides, the average value of the nodes from each side was used. For quantification of signal enhancement, tissue background signals were subtracted using the mean value from three uninjected mice with the same image acquisition settings.

### In vivo imaging through the skull

After i.c.v. tracer infusion, the mouse was transferred to the microscope and the head fixed in a stereotactic frame on a heating pad (37 °C). The skull was kept hydrated with warmed PBS under a glass coverslip, and dynamic imaging of the contralateral half of the brain was carried out for a maximum of 60 min after tracer infusion through the skull of the anesthetized mouse using a Zeiss StereoLumar.V12 stereomicroscope (as described above). Dynamic imaging through the skull was carried out with a gain of 300 and an exposure time of 50 ms (Cy5 filter for P40D680 tracer) or 200 ms (ICG filter for P40D800 tracer). In some cases, imaging was continued during administration of an overdose of 400 mg/kg ketamine and 1 mg/kg medetomidine (i.p.), or after an overdose with ketamine/medetomidine followed by transcardiac perfusion with ice-cold PBS.

For determination of CSF dynamics, circular ROIs of 1 mm diameter were placed over the location of the contralateral lateral ventricle and over the location of the quadrigeminal cistern in the acquired video using ImageJ/FIJI. Signal intensity within these ROIs was determined over time.

### Magnetic resonance imaging

Animals were examined in a horizontal-bore 9.4 T animal scanner (BioSpec Avance III 94/20; Bruker Biospin GmbH, Ettlingen, Germany) with a BGA12S gradient system with ParaVision 6.0.1 (Bruker Biospin GmbH) and a linearly polarized coil with an inner diameter of 40 mm (Bruker Biospin GmbH). Imaging was performed with a three-dimensional time of flight gradient recalled echo sequence (3D-TOF-GRE) originally adapted for imaging of peripheral lymph vessels [41] with recovery time of 12.0 ms, echo time of 2.5 ms, flip angle 25^o^, matrix 600 × 400 × 180, field of view 36.00 mm × 25.92 mm × 18.00 mm, zero fill 2, 1 average and a scan time of 4 min 19 s 200 ms. Signal intensity (SI) reduction in blood vessels was achieved by placement of a saturation slice over the mouse heart.

Three-dimensional maximum intensity projection (MIP) reconstructions, SI and noise measurements were performed with ParaVision 6.0.1 (Bruker Biospin GmbH). For SI analysis, ROIs were created manually in a single slice per measurement, 1 mm dorsal to the circle of Willis. For SI measurements in cortical parenchyma and noise determination from background SI, identically sized circular ROIs were employed, while cortical tissue containing vessels was segmented manually (see Supplemental Fig. 4).

For animals in which injection of MRI contrast medium was not successful (wrong location of the injection or significant backflow from the injection site), MR imaging experiments were discontinued.

### Brain sections

Mouse brains were dissected and fixed in 4% PFA at 4 °C for 48 h. A section of the brain between 1 mm rostral and 1 mm caudal to the needle insertion site was cut out by razor blade. Coronal sections were made from dorsal to ventral side at a thickness of 100 µm with a vibratome (Leica VT1000 S). Images were acquired with a Zeiss AxioZoom V16 microscope and ZEN 2 software.

### Analysis of tracer influx into the brain parenchyma

The percentage area of tracer coverage in brain sections was determined using ImageJ/FIJI as follows. The cerebral cortex was selected as an ROI on images of coronal brain sections. A uniform threshold was applied to all images, and background signal manually erased. The percentage area coverage of the signal within the ROI was determined. Analysis of all images was independently carried out in a blinded manner by two investigators, and an average was taken of six coronal sections per mouse.

The deepest tracer penetration within the dorsal cerebral cortex was determined using lines drawn perpendicular to the brain surface with ImageJ/FIJI. The two largest values were taken for the injected and contralateral sides, and an average of these four values was taken for six sections per mouse by two independent investigators. Due to clear differences between images from perfused and non-perfused brains, blinding was not feasible for this analysis.

### Statistics

Mice were randomly allocated to different experimental groups. Group sizes were estimated based on pilot studies to determine the success rate and reproducibility of the intraventricular infusions. All data are presented as mean ± SD. Means of two groups were compared using an unpaired two-tailed Student’s *t* test. Means of three groups were compared with one-way ANOVA with the Tukey’s multiple comparison post hoc test. Correlation analysis was done using Pearson’s correlation. All analyses were performed using GraphPad Prism V5.0 (GraphPad Software, San Diego, CA) and *p *< 0.05 was accepted as statistically significant.

## Results

### Outflow of CSF increases during awake conditions

**Fig. 1:**
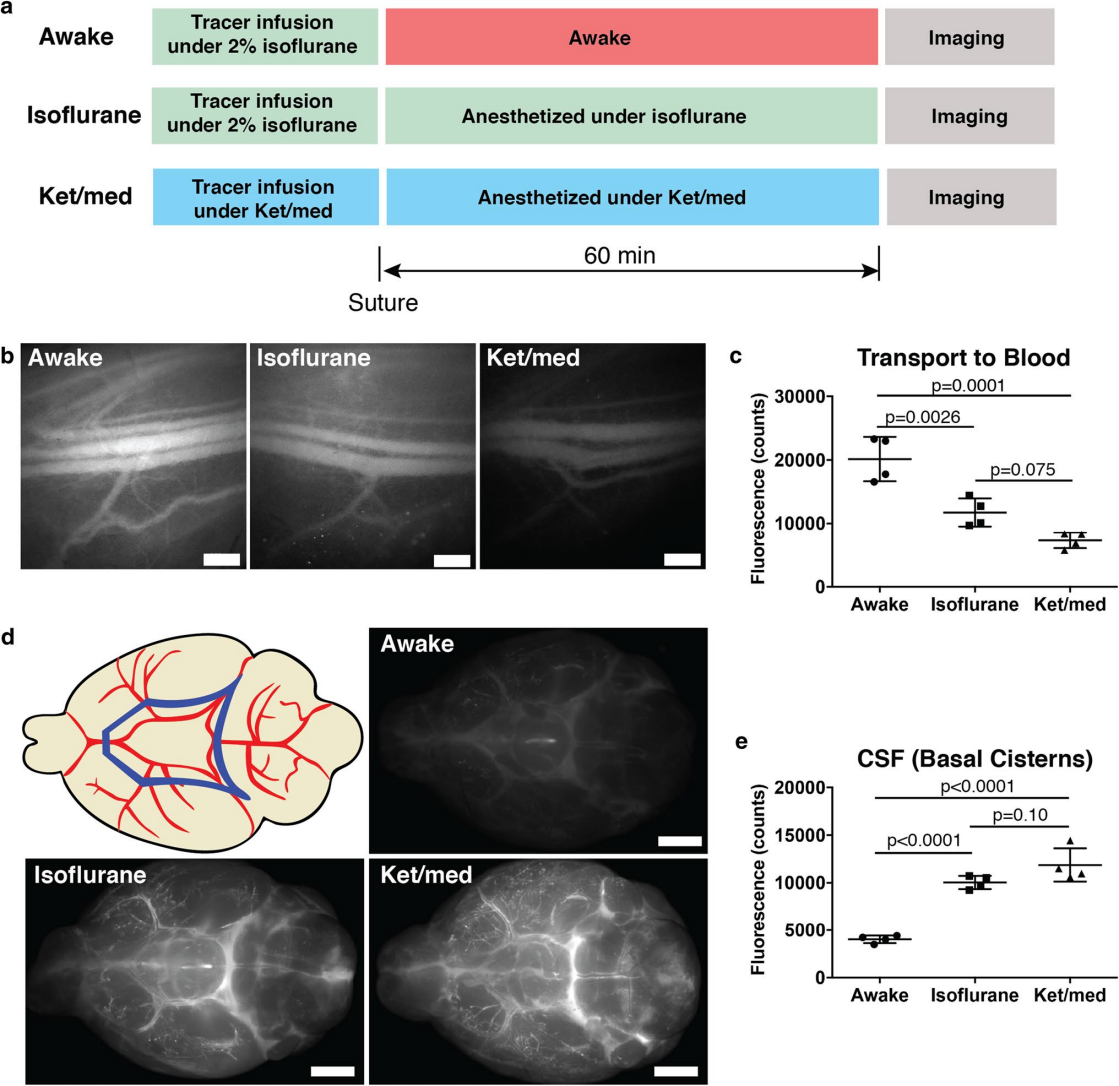
CSF outflow is increased during awake conditions. **a** Scheme of experimental design for quantification of CSF outflow during awake or anesthesia conditions. Before imaging at the saphenous vein, mice from awake and isoflurane group were given 80 mg/kg ketamine, 0.2 mg/kg medetomidine intraperitoneally. After imaging, all mice were overdosed with 400 mg/kg ketamine, 1 mg/kg medetomidine. **b** Representative images of saphenous bundle of blood vessels at 60 min post-infusion of P40D680 into lateral ventricle. Scale bar: 500 µm. **c** Quantification of P40D680 signal in the blood 60 min after tracer i.c.v. infusion. **d** Scheme indicating ROI and ex vivo imaging of the basal aspect of the brain at 60 min post-infusion showing presence of P40D680 tracer at circle of Willis and cisterns. Blue polygon in upper left image represents the analyzed ROI. Scale bar: 2000 µm. **e** Quantification of P40D680 signal at basal cisterns 60 min after infusion

Using dynamic near-infrared fluorescence imaging, we have previously demonstrated that CSF-infused tracers reach the systemic circulation in anesthetized mice by transport through the lymphatic system rather than via a direct outflow from the subarachnoid space to venous blood [37]. With this in mind, we first aimed to determine the levels of CSF-injected tracer that reached the systemic blood in mice that were allowed to wake after i.c.v. infusion versus mice that were maintained under anesthesia. As shown in the experimental design scheme in Fig. 1a, three groups of mice were assessed for CSF transport to blood at 60 min after lateral ventricle infusion of a 40 kDa bulk flow tracer [37]. Two groups of mice were infused with 2.5 µL of 200 µM P40D680 over the course of 2.5 min under isoflurane anesthesia. After suture of the skin above the skull, mice in the awake group were allowed to recover and move normally within the cage, while mice in the isoflurane group were maintained on a heating pad under isoflurane. At 60 min after the i.c.v. infusion, both groups of mice were injected with ketamine/medetomidine (ket/med) for in vivo imaging of tracer signal within the saphenous vein. A third group (ket/med group) was kept under ket/med anesthesia for the duration of the infusion, maintenance and imaging procedures.

Using a fluorescence stereomicroscope, imaging of P40D680 signal within the saphenous vein indicated that mice from the awake group had significantly increased tracer levels within the systemic blood at 60 min compared to mice that were maintained under either anesthetized condition (Fig. 1b-c). The tracer levels at 60 min in awake mice corresponded to 62.9 ± 10.9% of the i.c.v infused tracer present within the blood when compared to the fluorescent signals in mice that were intravenously injected with the same dose (20,149 ± 3490 vs. 32,018 ± 718 counts). After killing the mice via ket/med overdose and harvesting the brains, we compared the signals of P40D680 that remained at the basal cisterns around the circle of Willis as another measure of CSF clearance (Fig. 1d). As expected, mice in the awake group had significantly less P40D680 remaining at 60 min compared to mice that were under either type of anesthesia (Fig. 1e). These results indicate that clearance of CSF-infused tracers is increased during awake conditions, consistent with our findings of enhanced lymphatic clearance during awake conditions in other regions of the body [44].

### Dynamics of CSF outflow support lymphatic efflux

**Fig. 2:**
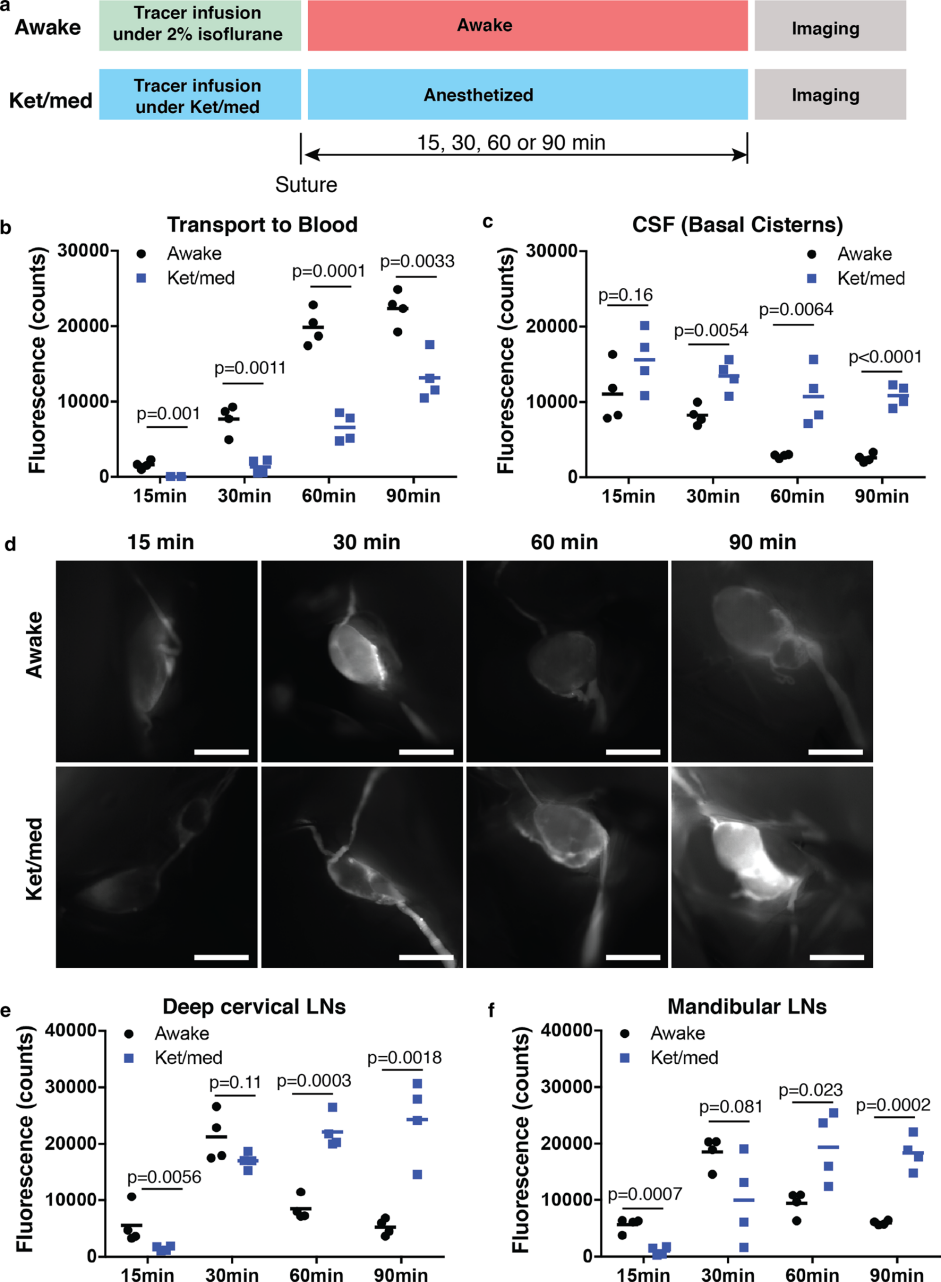
Dynamics of outflow support CSF drainage through lymphatic vessels**. a** Scheme of experimental design for quantification of the dynamics of CSF outflow during awake or ket/med anesthesia conditions. **b** Quantification of P40D680 signals in the blood. **c** Quantification of P40D680 signals at basal cisterns. **d** Representative in situ images of deep cervical LNs at 15, 30, 60 and 90 min post-infusion of P40D680 into lateral ventricle. Scale bars: 1000 µm. **e** Quantification of P40D680 signals in the deep cervical lymph nodes (LNs). **f** Quantification of P40D680 signals in the mandibular LNs

Since a significant portion of the injected dose quickly reaches the systemic blood in the awake mice, we aimed to provide support that CSF outflow occurs through lymphatic vessels under these conditions. As live monitoring of the dynamics of tracer transport to systemic blood in awake mice is not feasible, we killed mice at four different time points (15, 30, 60 and 90 min) after i.c.v. infusion to assess tracer levels in the blood, the basal cisterns and the CNS-draining lymph nodes. We compared the signals in mice that were allowed to wake to signals from mice that had been infused and maintained under ket/med (Fig. 2a), conditions where we have previously demonstrated that CSF tracer outflow occurs predominantly through a lymphatic route [37].

At 15 min after i.c.v. infusion, tracers were already present at low levels in systemic blood in the awake group, but the signals were not above background levels in the ket/med group (Fig. 2b). The signals in blood increased over time in both groups and were 5.7-fold higher at 30 min, 3.0-fold higher at 60 min and 1.8-fold higher at 90 min in awake mice compared to ket/med mice. The signal intensity at the basal cisterns showed an opposite pattern with significantly lower levels of tracer remaining at 30, 60 and 90 min in the awake group (Fig. 2c). Intensity of the signals in the CNS-draining deep cervical and mandibular lymph nodes imaged in situ at 15 min showed higher levels in the awake group; however, no significant differences between the two groups were apparent at 30 min (Fig. 2d–f). At 60 and 90 min, the levels of tracer in both deep cervical and mandibular lymph nodes in awake mice were significantly lower than those in ket/med mice, consistent with the observation that most of the infused tracer in the awake mice had already reached the systemic blood.

As further evidence for lymphatic clearance of CSF under awake conditions, we next aimed to demonstrate a time point shortly after recovery from anesthesia at which signals could be found within the lymphatic system but not yet in the systemic blood. This was challenging as blood signals were already elevated at the 15 min time point in the awake group, as shown in Fig. 2b. We speculated that a reduced infusion rate and volume may slow down the dynamics of CSF outflow, which has been shown to be dependent on intracranial pressure [9, 23, 39]. To test this, we infused into the lateral ventricle identical doses of P40D680 but at different volumes and rates. This led to clear effects on the transport to blood with a delay in the transport time when the lowest volume was infused (Supplementary Fig. 1a, b). Therefore, we infused 1 µL of 500 µM P40D680 and killed the mice at 10 min after i.c.v. infusion, shortly after the mice had awakened (Supplementary Fig. 1c). As shown in the representative mouse in Supplementary Fig. 1d, using this protocol we could not detect tracer within the bloodstream (above the assay sensitivity of 0.2% of the infused dose [37]), but found that tracer was readily detected in the collecting lymphatic vessels and draining lymph nodes by 10 min after tracer infusion (Supplementary Fig. 1e). In sum, these dynamic imaging results support the conclusion that CSF clearance occurs rapidly through lymphatic vessels in mice during awake conditions.

### Distribution of tracers to the PVS is inversely correlated with CSF outflow

**Fig. 3:**
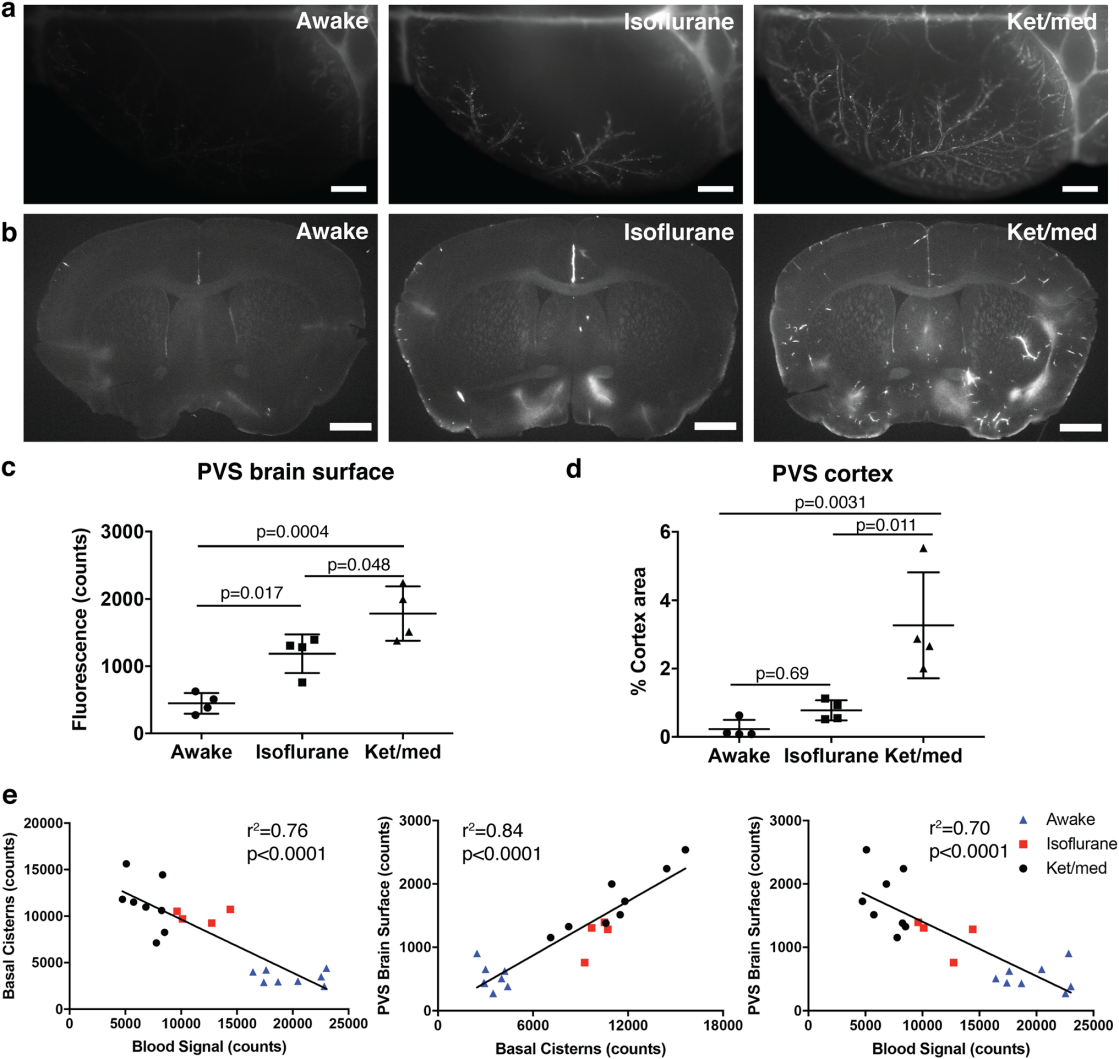
Spread to paravascular space (PVS) is inversely correlated to CSF outflow. **a** Representative images of tracer-filled PVS on the brain surface of the dorsal hemisphere contralateral to the infusion site. Scale bars: 1000 µm. **b** Representative images of tracer accumulation in PVS around penetrating vessels on 100 µm brain sections. Scale bars: 1000 µm. **c** Quantification of P40D680 signal on the brain surface 60 min after infusion. **d** Quantification of P40D680 signal in the cortex of the 100 µm brain sections 60 min after infusion. **e** Correlations from all mice examined at 60 min demonstrating relationships between signals at basal cisterns, brain surface and systemic blood. All quantifications are normalized to signals in *n* = 3 uninjected mice

Glymphatic (paravascular) influx of CSF-injected tracers into the brain has been found to be reduced in awake mice compared to those under sleeping or anesthetized conditions [54]. Therefore, we next assessed whether the rapid tracer efflux from the CSF that we observed during awake conditions could affect the spread of the tracers to the PVS of the brain surface and the parenchyma of the brain. Similar to the levels of tracer intensity demonstrated in the basal cisterns (Fig. 1d), there were obvious differences in the amount of tracer located within PVS on the dorsal brain surface and parenchyma of the brain (Fig. 3a, b). Mice anesthetized with ket/med demonstrated an increased signal intensity on the surface of the brain, with a clear PVS pattern observed, compared to mice that were awake (Fig. 3c). Mice under isoflurane anesthesia showed an intermediate pattern of tracer spread. The PVS within the brain parenchyma also presented with significantly increased tracer signal in the ket/med group compared to mice in the isoflurane and awake groups (Fig. 3d).

We then pooled the data from mice analyzed under all three conditions at the 60 min time point and performed correlation analyses to determine whether there were any significant relationships between the tracer levels within the blood, at the basal cisterns and within the PVS of the brain surface. These analyses showed clearly that the spread of tracer to the PVS of the brain surface was directly related to the amount of tracer remaining at the basal cisterns and indirectly proportional to the amount that had reached the systemic blood (Fig. 3e). These findings support the concept in which rapid outflow of CSF-administered tracers observed during awake conditions limits the distribution of tracers to the PVS (Supplementary Movie 1).

### Tracers accumulated within PVS of the brain surface around both arteries and veins, even at early time points

Close examination of the surface of the brain at 60 min indicated that tracers were found not only along brain surface arteries, but also along veins (Supplementary Fig. 2a). We confirmed this using a SMMHC-GFP reporter mouse where contractile arterial smooth muscle cells (SMC) show green fluorescence. The tracer was localized outside the SMC layers of the arteries (Supplementary Fig. 2b), consistent with a “paravascular” rather than a “perivascular” or “intramural” location as defined by Engelhardt et al. [18]. Surprisingly, even in mice that were killed only 2 min after the completion of the infusion into the ventricles, we were able to detect the presence of tracer along branches of the caudal rhinal veins (Supplementary Fig. 2c). In these mice, there was no obvious tracer penetration into the parenchyma; therefore, the tracer spread from PVS of arteries to veins did not appear to be occurring within the brain (Supplementary Fig. 2d). These results indicate that pathways for CSF flow may exist between the PVS of arteries and veins at the brain surface.

### Through-skull in vivo imaging demonstrates spread of tracers within the PVS of large-caliber vessels of the brain surface

**Fig. 4:**
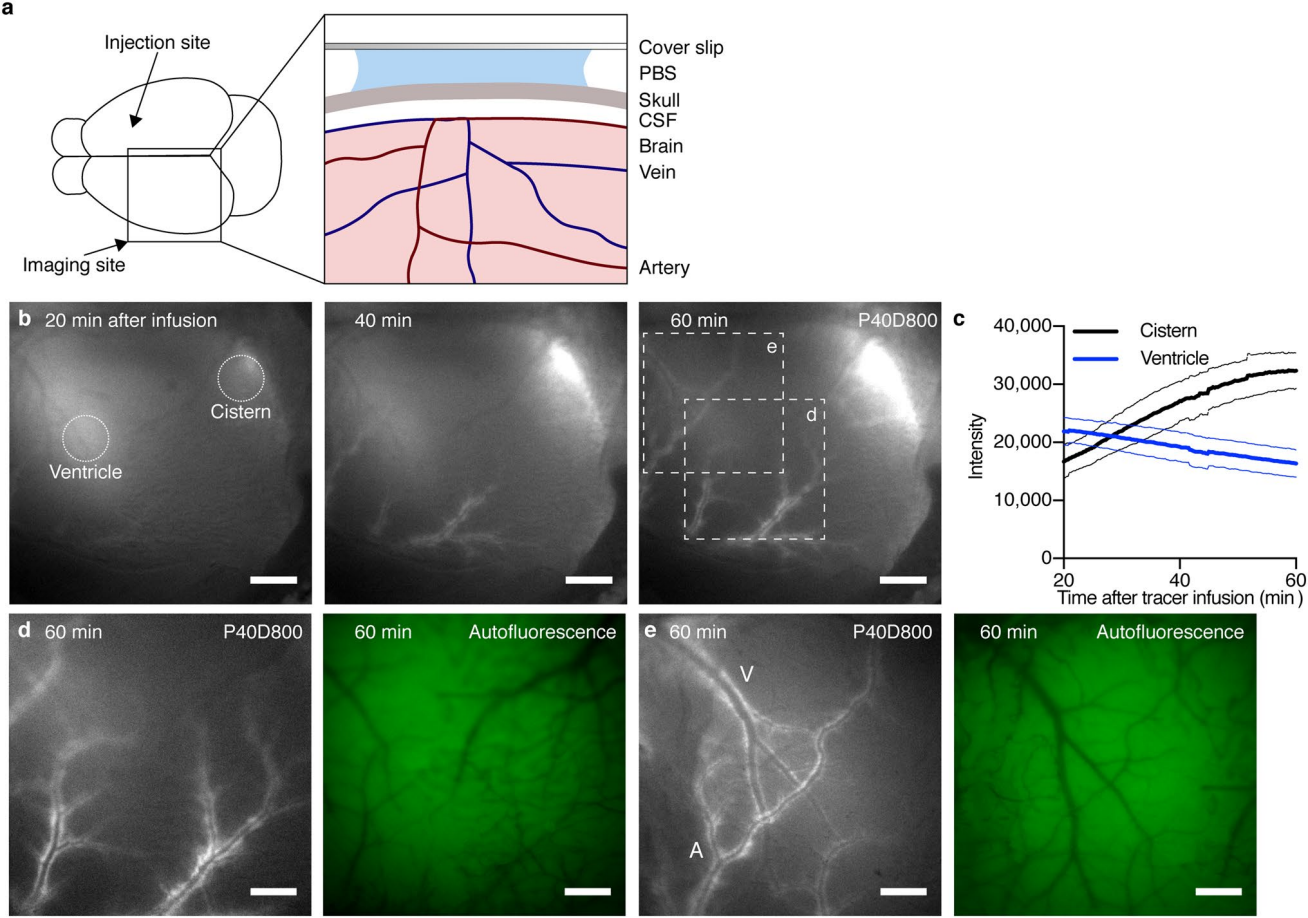
Tracer spread from the CSF is limited to PVS of surface blood vessels in vivo. **a** Schematic illustration of setup for in vivo near-infrared imaging through the skull. **b** Representative images of tracer spread along brain surface blood vessels visualized through the skull 20, 40 and 60 min after infusion of 2.5 µL 200 μM P40D800 into the contralateral ventricle. Scale bars: 1000 μm. Dotted circles show 1 mm diameter ROIs used for quantification of tracer signal from lateral ventricle and quadrigeminal cistern. **c** Quantification of fluorescence signal from the lateral ventricle (blue) and quadrigeminal cistern (black) after tracer infusion as in (**b**) (*n* = 5). Data show mean (solid lines) ± SD (dashed lines). **d–e** Close-ups of region shown in (**b**) with corresponding autofluorescence images 60 min after tracer infusion indicating tracer spread along large surface arteries (A) and transfer to surface veins (V). Scale bars: 500 μm

We hypothesized that the spread of tracers within the PVS of the brain surface in mice anesthetized with ket/med might be demonstrated in vivo using NIR imaging through the intact skull. To establish this assay, we infused P40D800 tracer into the right lateral ventricle and imaged over the left cortical hemisphere through the skull of the mouse with a NIR-sensitive fluorescence stereomicroscope (Fig. 4a). The longer wavelength P40D800 tracer exhibits similar properties to the P40D680 tracer but allows NIR imaging with better depth penetration and lower tissue autofluorescence. When imaging was initiated at 20 min post i.c.v. infusion, tracer signals emanating from the lateral ventricles and cisternal spaces of CSF, such as the quadrigeminal cistern, were seen (Fig. 4b). Quantification of videos of these regions over time showed an expected loss of signal from the ventricles concurrent with an increasing signal from the CSF in the quadrigeminal cistern (Supplementary Movie 2, Fig. 4c). During this time, tracer signals became apparent along the middle cerebral artery (MCA) and its branches and slowly spread within the network of PVS around these vessels (Fig. 4b, d). In some mice, we could observe a spread of tracer from the PVS of the branches of the MCA to some of the larger veins such as the dorsal middle cerebral and dorsal rostral cerebral veins (Supplementary Movie 3, Fig. 4e). However, at no point in time was it obvious that there was a spread of tracer from the larger brain surface arteries to the penetrating arteries entering the cortex. Therefore, we conclude that under our in vivo experimental conditions, the spread of tracer over the dorsal hemisphere is confined to the large caliber arteries and veins at the brain surface.

### Rapid paravascular spread of tracers into the brain parenchyma after death

Our imaging experiments revealed discrepancies between in vivo and ex vivo patterns of tracer within the PVS. In vivo, the tracer-filled spaces appeared wider [5], there was a more limited spread to the PVS over the dorsal hemispheres and there was no obvious penetration of tracers along cortical blood vessels despite being clearly evident on brain sections (Fig. 3b). Therefore, we speculated that a post-mortem artifact might be present that could explain these differences.

**Fig. 5:**
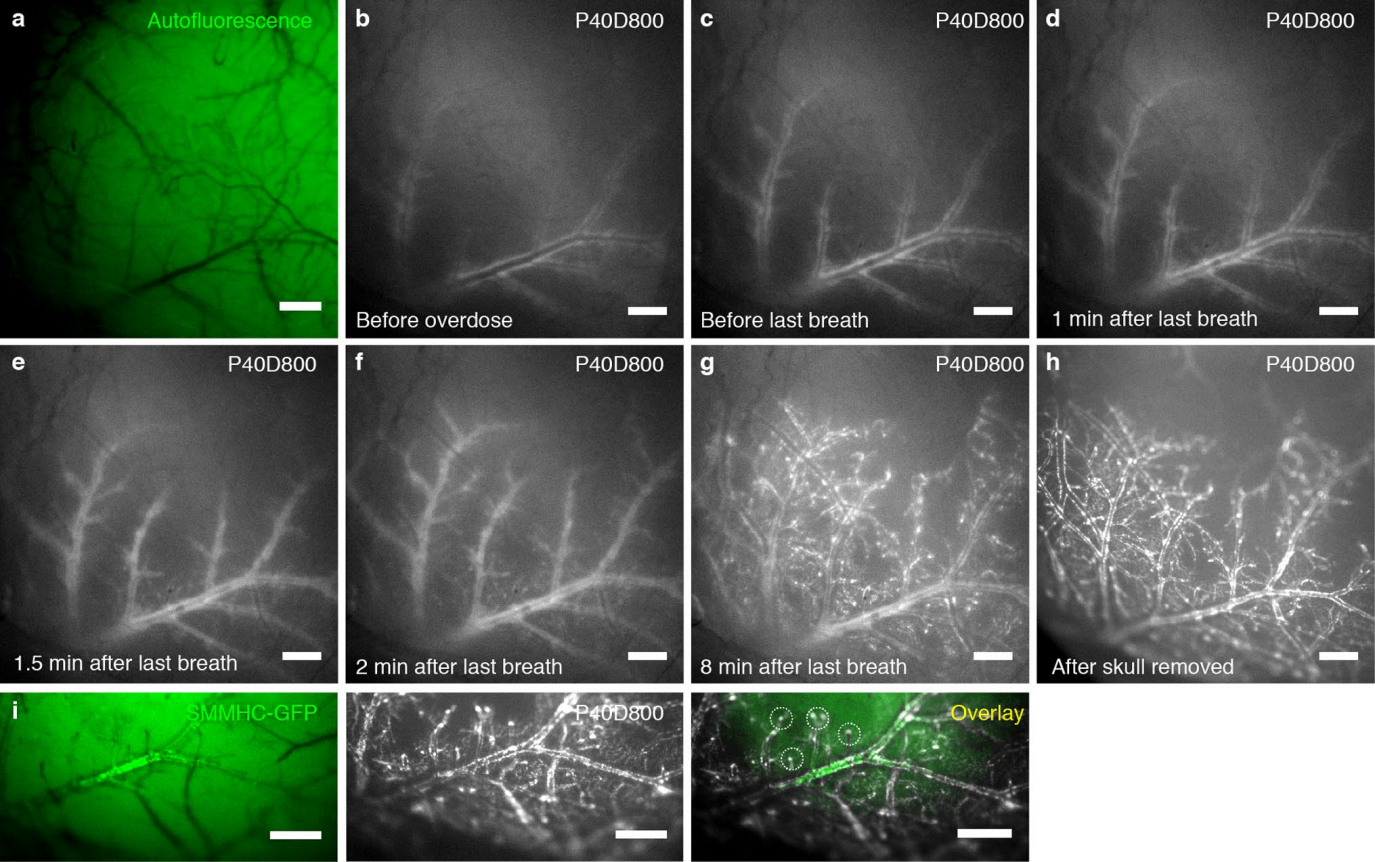
Extensive post-mortem tracer spread on brain surface and to penetrating blood vessels. **a**–**h** Images of tracer spread visualized after infusion of 2.5 µL 200 μM P40D800 into the contralateral ventricle. Imaging through the skull (**a**–**g**) or after skull removal (**h**). **a** Autofluorescence image of brain region imaged. **b** Tracer spread 60 min after infusion of 2.5 µL 200 μM P40D800 into the contralateral ventricle. **c** Tracer spread after overdose with ket/med i.p. but before last breath. **d** Tracer spread 1 min after last breath. **e** Tracer spread 1.5 min after last breath suggesting constriction of arteries initiating from the MCA. **f** Tracer spread 2 min after last breath showing spreading of arterial constriction to smaller branches of the MCA. **g** Tracer spread 8 min after last breath showing spreading to penetrating blood vessels. **h** Imaging of tracer spread after skull removal indicating that signal detection was not significantly obstructed by imaging through the skull. **i** Images of tracer spread in brain of SMMHC-GFP mouse ex vivo 60 min after infusion of 2.5 µL 200 μM P40D800 into the lateral ventricle. SMMHC-GFP positive vessels (green) mark arteries with contractile smooth muscle cells. Circles indicate location of tracer (white) at penetrating blood vessels. Scale bars: 500 μm

To test this, we acquired videos through the skull of mice before and during an overdose with ket/med. Mice were imaged in vivo until 60 min after tracer infusion, at which point the tracer spread was limited to the larger surface vessels, especially the MCA (Fig. 5a, b, Supplementary Movie 4). We then overdosed the animal via i.p. injection of a lethal dose of ket/med. During the next few minutes, there was a minimal further spread of tracer while the animal exhibited gasping activity (Fig. 5c). Immediately after the final breath was taken, a wave was seen to spread through the cortex that originated laterally with vasoconstriction of the large branches of the MCA and spread medio-dorsally over the surface of the cortical hemisphere (Supplementary Movie 4). Following this wave, there was an advance of tracer towards the smaller branches of the MCA and the penetrating arteries, as well as to the venous PVS (Fig. 5d–g). Comparison of the images from the same mice before and after skull removal clearly demonstrated that the images acquired through the skull revealed the true extent of tracer spread (Fig. 5h). Notably, tracer signal at the penetrating vessels was only apparent after death (Fig. 5i).

**Fig. 6:**
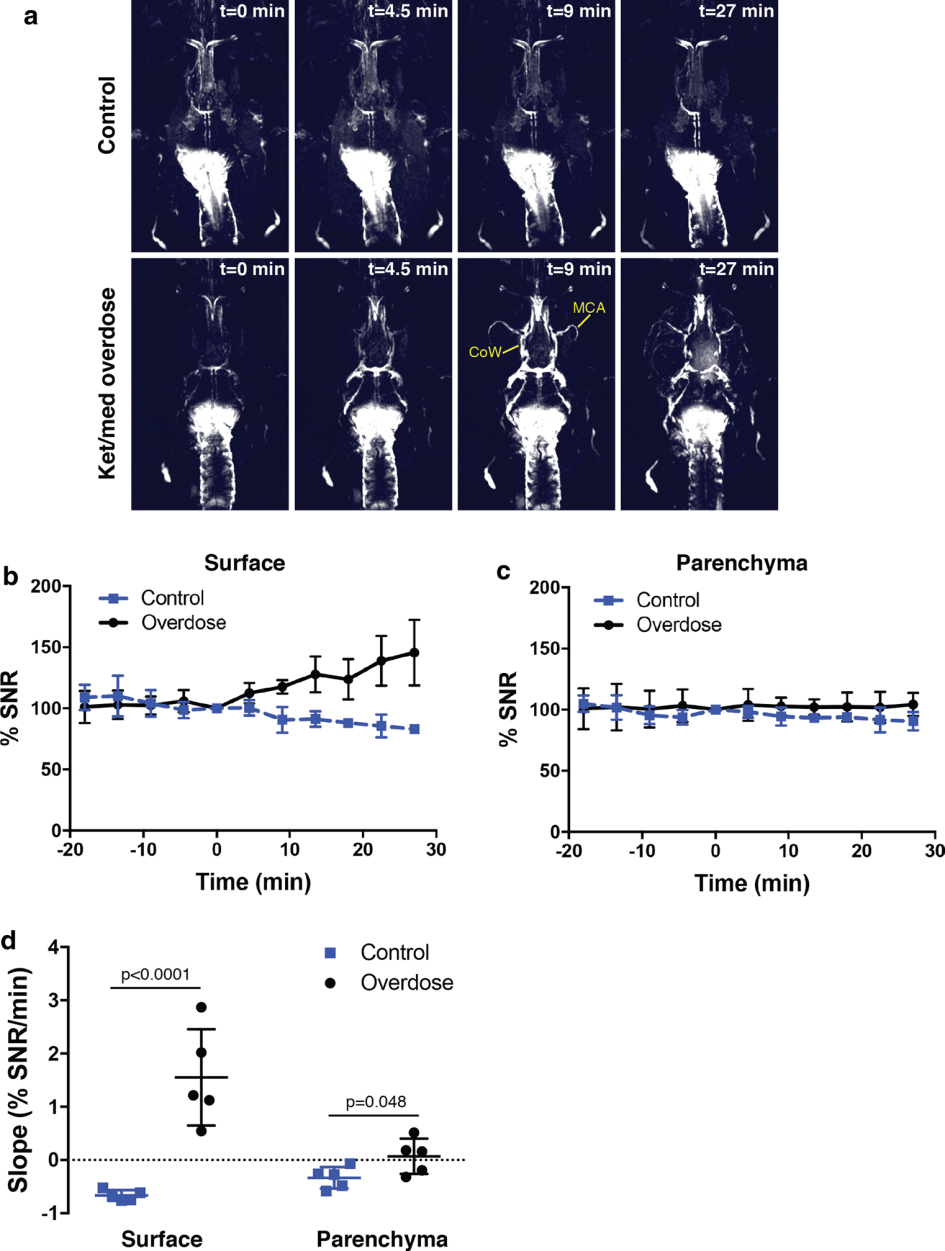
Spread of contrast agent to PVS after death as detected with MRI. **a** Visualization of tracer spread after infusion of 5 µL of a Gadospin D solution at 25 mM gadolinium into the cisterna magna; data acquired with a series of T1-weighted MRI measurements (three-dimensional time of flight gradient recalled echo sequence). MIP images on the upper panel show a mouse that was kept alive under ket/med anesthesia and showed no spread of the tracer towards the PVS. The bottom panel images show a mouse that was overdosed with ket/med; the time of death is indicated as *t* = 0. Strong enhancements of signal are detectable 9 min after death at the circle of Willis (CoW) and at the middle cerebral artery (MCA). Smaller branches of the MCA are clearly visible 27 min after death. **b**, **c** Quantification of signal-to-noise ratio (SNR) after tracer infusion in the cisterna magna in mice kept under ket/med anesthesia (*n* = 5) or in mice killed by ket/med overdose (*n* = 5). Regions of interests were defined on the surface of the cortex (**b**) and the cortical parenchyma (**c**). SNR values were normalized to the time of death and set to time = 0. Data show mean ± SD. **d** Comparison of the slopes calculated by linear regression of SNR over time for the surface of the cortex and for cortical parenchyma in the control group and in the group of mice killed by ket/med overdose

We next confirmed that an influx to the PVS was occurring after death using MRI. Infusions of a macromolecular contrast agent, Gadospin D, were performed into either the cisterna magna or the right lateral ventricle and a series of three-dimensional MRI datasets was then acquired with a T1-weighted sequence. After a period of approximately 40 min, one group of mice (*n* = 5) was overdosed with ket/med while a control group (*n* = 5) was maintained under ket/med anesthesia. Clear enhancements of signal occurred at the circle of Willis and the MCA after death that was not apparent in the control groups (Fig. 6a, Supplementary Fig. 3, Supplementary Movies 5 and 6). Next, we performed quantification in mice that were infused through the cisterna magna since they did not have interfering signals from the ventricles. Signal-to-noise ratio measurements were made using ROIs on the surface of the cortex (assumed to be PVS) and the cortical parenchyma (Supplementary Fig. 4). After normalization of the signals to the time of death (set to time = 0), these assessments revealed that the slope of the signal increase was significantly greater in both regions after death when compared to the control mice (Fig. 6b–d). Importantly, no detectable influx of contrast agent occurred in the parenchyma of the in vivo control group at any point after infusion.

We also tested whether cardioperfusion would limit the spread of tracers to the parenchyma at the time of death. Videos acquired immediately after the completion of perfusion demonstrated that the spread of tracers was also occurring post-mortem under these conditions (Supplementary Movie 7). Assessment of the influx depth of tracer spread in the PVS of the parenchyma demonstrated no difference between mice killed by overdose or via cardioperfusion (Supplementary Fig. 5). We conclude that shortly after death a significant spread of tracer occurs through the PVS of the larger surface vessels to the PVS of the parenchyma.

## Discussion

In this study, we demonstrated using intraventricular infusions of a bulk flow tracer and fluorescence stereomicroscopy that CSF outflow was increased in awake mice compared to anesthetized mice. Evaluation of the dynamics of this outflow supports the conclusion that CSF clearance occurs through the lymphatic system in this species. An inverse relationship was found between the efflux of CSF to the systemic blood and the spread of CSF tracers to the paravascular spaces as assessed ex vivo. In vivo imaging of PVS spread of tracers at the brain surface demonstrated that this spread was limited to the PVS of larger caliber arteries and veins. Finally, we found that a rapid influx of tracers to the PVS of the cortical parenchyma occurred just after the death of the animal.

A substantial increase in the outflow of CSF tracers to the systemic blood in awake mice was found compared to mice that were anesthetized. There is very little similar data to compare to regarding this point, although one previous report demonstrated increased CSF clearance of radiolabeled tracer from the spine in active compared to resting subjects [17]. It is likely that physical activity affects CSF pressure and increases flow towards efflux pathways. Respiration and arterial pulse are driving forces for CSF flow and mixing [16, 20] and the rates of both are naturally higher in awake versus anesthetized animals. These results also lend further support for a CSF outflow pathway that is predominantly lymphatic in nature [9, 37]. Lymphatic clearance is highly dependent on muscular activity and transport through lymphatic vessels is dramatically increased when the subject is awake [22, 44, 49]. The concept of lymphatic rather than venous outflow of CSF still remains to be rigorously evaluated in humans, however, some evidence does exist to support this pathway in non-human primates and cadavers [31, 38].

An increased CSF outflow while awake is not fully consistent with the concept of increased CSF flushing through the brain during sleep or deeply anesthetized conditions [54]. In the glymphatic model, CSF within the arterial PVS serves as an input fluid to the brain. If the CSF turnover is rapid during awake conditions, this would limit access of CSF-infused tracers to the PVS, a factor that was not considered during the original study [25, 54]. Another recent MRI study has challenged the concept that increased glymphatic flow occurs in anesthetized mice compared to awake mice [21]. However, this study concluded that CSF influx was actually increased during awake conditions, which we have not found to be the case. It is unclear whether the tracer that was measured in the brain by MRI in vivo was intravascular after effluxing the CSF to reach the systemic circulation rather than a direct influx into the parenchyma from CSF as proposed by the authors. In addition, the low-molecular weight tracer used in this study as well as other recent MRI studies [7, 34, 47] would exhibit significant concentration gradient-dependent diffusion into the brain parenchyma and thus would not accurately demonstrate the bulk flow pathways of CSF.

It is important to note that it was not our aim to assess sleeping, unanesthetized mice with our methods, nor have we monitored clearance of tracer injected into the parenchyma. Therefore, theoretically, an increased clearance of metabolites from the brain might indeed occur under sleeping conditions. The destination for the efflux fluid in the glymphatic model is not exactly clear; one version of the model shows fluid exiting through the PVS around veins to reach the CSF while another proposes that there may be connections to lymphatic vessels within the dura mater [4, 28, 35, 36]. However, we were unable to demonstrate any paravascular influx of CSF into the brain during anesthetized in vivo conditions and we have found significantly increased clearance of CSF under waking conditions. Therefore, at this point, it is difficult to imagine how an increased brain flushing during sleep via a bulk CSF influx through the glymphatic system could be occurring as originally envisaged.

The close correlation between signals in the basal cisterns and the PVS of the brain surface highlights the circle of Willis as an important potential access point for CSF and its components into the PVS. The circle of Willis is the main source of the arteries that feed into the brain. A recent study in rats has confirmed an earlier observation that stomata exist in the pia mater covering these arteries which may provide anatomical pathways for CSF and macromolecules from the basal cisterns to the PVS [42, 56]. The major CSF outflow routes along several cranial nerves [37] (e.g., olfactory, optic, trigeminal) are located immediately rostral to the circle of Willis, indicating that CSF flow could be directed either out of the skull or into the arterial PVS depending on the physiological conditions.

Our data support the concept that some portion of CSF can spread to the brain surface PVS in vivo, at least under anesthetized conditions where there is reduced CSF outflow. We have also observed a spread of tracer from the PVS of arteries to the PVS of veins on the brain surface, consistent with the recently published concept of low resistance pathways for CSF flow at this location [6]. It will be interesting to examine at an anatomical level whether connections are present between the leptomeningeal sheaths of the PVS of arteries and veins. CSF may also spread along the large vessels that penetrate from the ventral aspect into the brain, such as into the midbrain as shown in MRI studies [15]. A recent MRI study in humans has shown that the diffusion of a low molecular weight contrast agent into the brain parenchyma correlated with the availability of the contrast agent in the surrounding CSF [47]. Thus, a dynamic balance between CSF turnover and CSF solute distribution to the brain may exist that could be altered under different physiological or pathological conditions.

The most surprising finding of this study was a major influx of tracer into the PVS that occurred immediately after the death of the animal. This observation was predicted by the prominent neuroscientist Lewis Weed who wrote in 1914 that “ordinarily, after death, cerebrospinal fluid is aspirated by the brain” [53]. One might expect that the substantial loss of blood pressure that occurs within the brain at death would establish a gradient for flow from the CSF to the brain parenchyma [53]. Thus, quantifications of tracer that are performed within the PVS or parenchyma of the brain ex vivo are likely overestimates of the levels that are present in vivo. This phenomenon may also account for some of the previous findings based on ex vivo assessments that have concluded that CSF influx was occurring into the brain within minutes of tracer administration [2, 8, 45, 52].

In sum, our data support the conclusion that a bulk flow of CSF into the brain along the PVS likely does not occur under physiological conditions and that the rate of outflow of CSF is a major factor determining whether substances within CSF can spread to the brain. This work may have important implications in drug delivery to the CNS after intrathecal administration and in several conditions that may lead to altered CSF flow dynamics such as traumatic brain injury and stroke.

## Electronic supplementary material

Below is the link to the electronic supplementary material.

Supplementary material 1 (PDF 6014 kb)

Supplementary material 2 (MOV 4449 kb)

Supplementary material 3 (MOV 11996 kb)

Supplementary material 4 (MOV 7204 kb)

Supplementary material 5 (MOV 10886 kb)

Supplementary material 6 (MOV 517 kb)

Supplementary material 7 (MOV 466 kb)

Supplementary material 8 (MOV 682 kb)
