# Supplementary material for: Rapid lymphatic efflux limits cerebrospinal fluid flow to the brain
Source: Acta Neuropathol. 2018 Oct 10;137(1):151–65. doi: 10.1007/s00401-018-1916-x (PMC6338719; doi:10.1007/s00401-018-1916-x)
Supplement: Supplementary file 1 — Supplementary material 1 (PDF 6014 kb) [file 401_2018_1916_MOESM1_ESM.pdf]

Extended Data

Supplementary figures

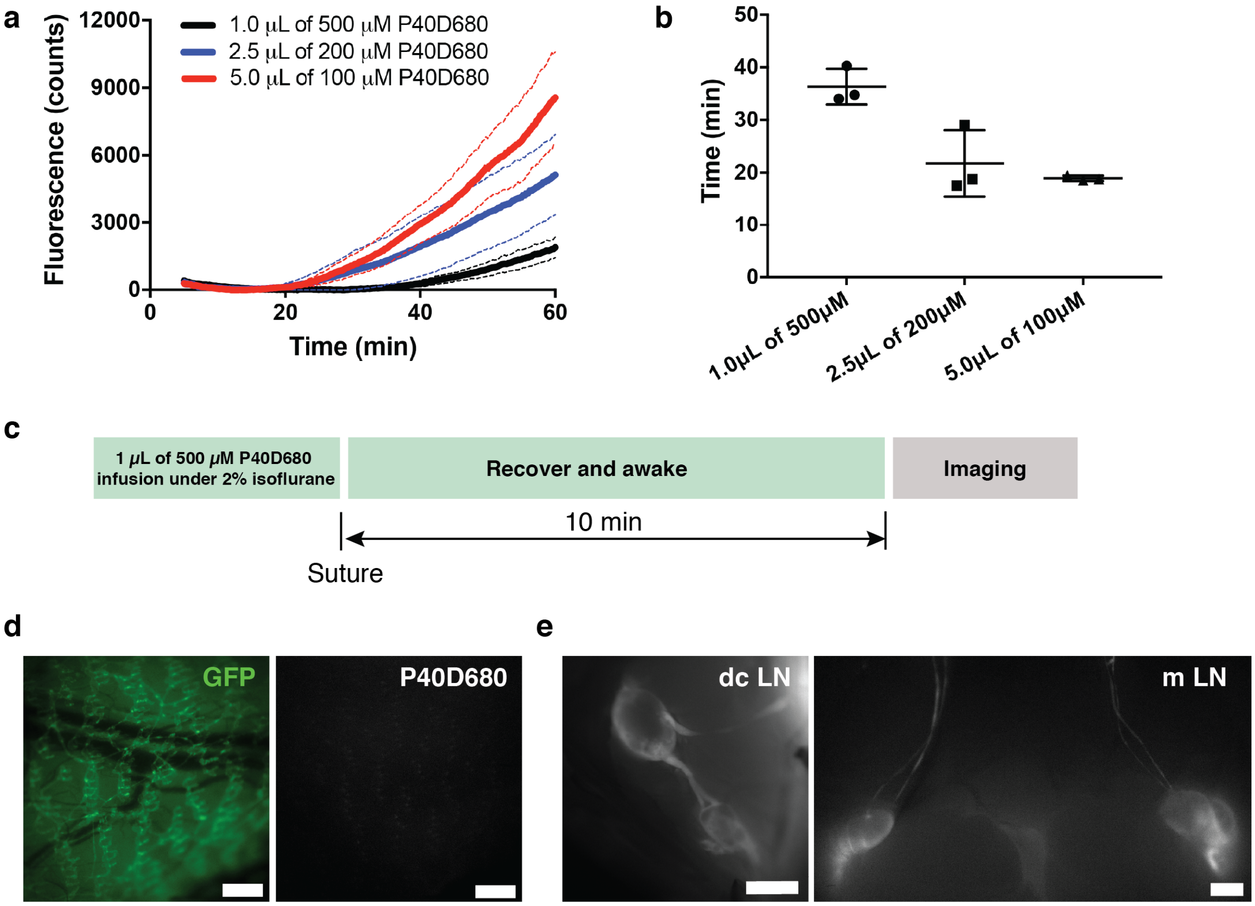

Fig. S1. Demonstration of a time point with presence of tracer in lymphatic system but not in systemic blood using a reduced infusion rate. a, Validation study of transport to blood assay with different volume infusions of the same dose of P40D680. Infusions were performed over 2.5 min at different rates. n=3, per group. Y-axis represents fluorescence signal enhancement normalized to baseline signal (lowest 10 consecutive measurements). Dashed lines indicate the SD. **b**, Quantification of transit time to the blood. **c,** Scheme of experimental design to demonstrate that there is a point in time at which signals are evident in the lymphatic system but not yet in systemic blood. Mouse was active for the final 2 min of the approximately 10 min

period between the end of the low-volume infusion and imaging. **d,** Representative images of P40D680 signal in the saphenous vein 10 min after infusion into lateral ventricle. Scale bars: 500 μm. **e,** Representative images of P40D680 signal in deep cervical LN (dc LN) and mandibular LN (m LN) 10 min after infusion into lateral ventricle. Scale bars: 1000 μm.

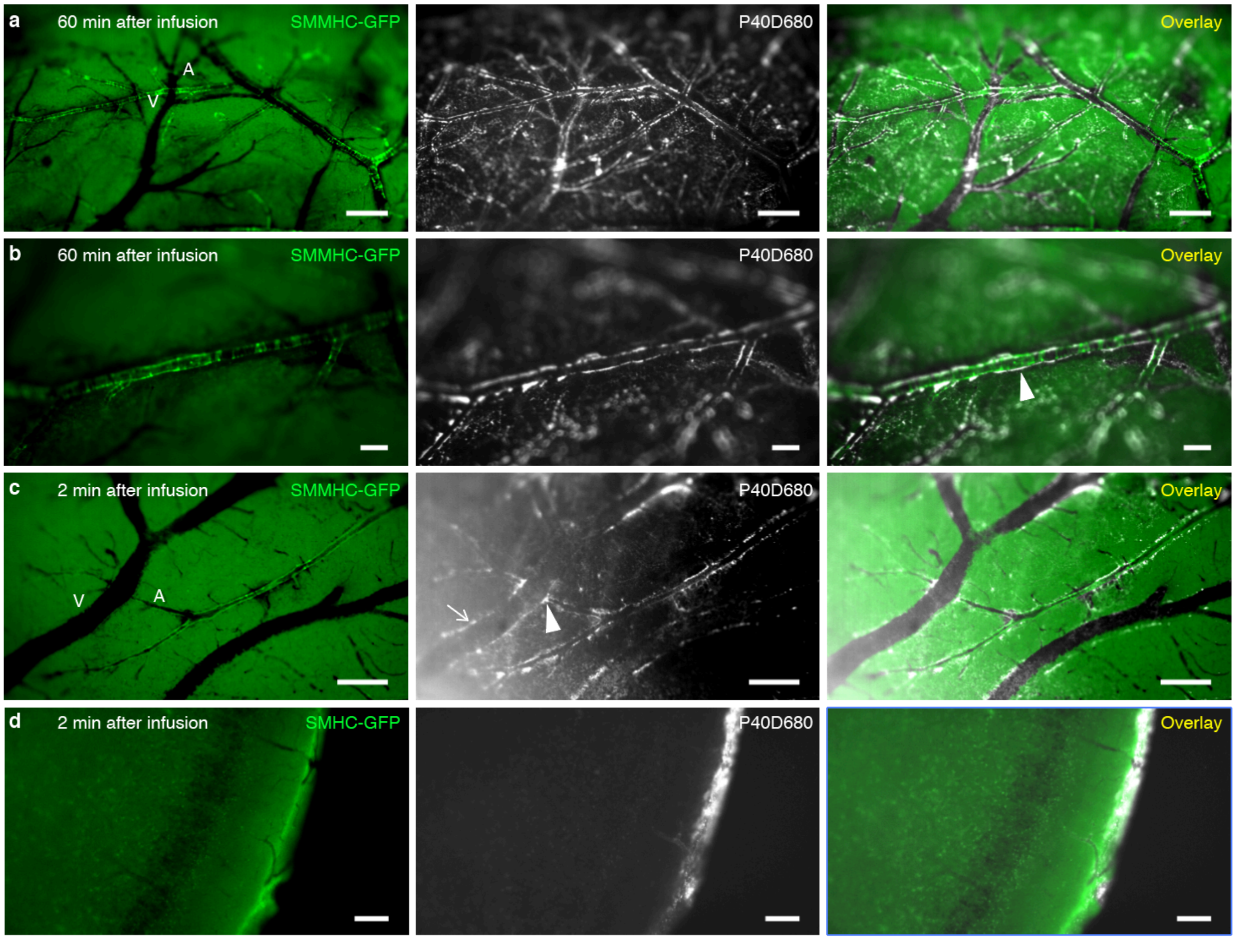

Fig. S2. Characterization of paravascular location of tracer and spread between arteries and veins at the brain surface. a-b, Ex vivo images of tracer spread 60 min after i.c.v. infusion of 2.5 µL 200 µM P40D680 in an SMMHC-GFP mouse suggesting spreading of tracer (white) between an artery (A) and a vein (V) at the brain surface (**a,** scale bars: 500 µm) and a location of tracer outside of the smooth muscle cell layer of the artery (**b,** SMMHC-GFP, green, scale bars: 100 µm). **c-d,** Ex vivo images of tracer spread 2 min after i.c.v. infusion of 2.5 µL 200 µM P40D680 in an SMMHC-GFP mouse indicate spreading between artery (A) and vein (V) is already occurring at this point at the brain surface (**c,** scale bars: 250 µm), while image of 100 µm brain section shows no tracer penetration into the brain parenchyma (**d,** scale bars: 100 µm).

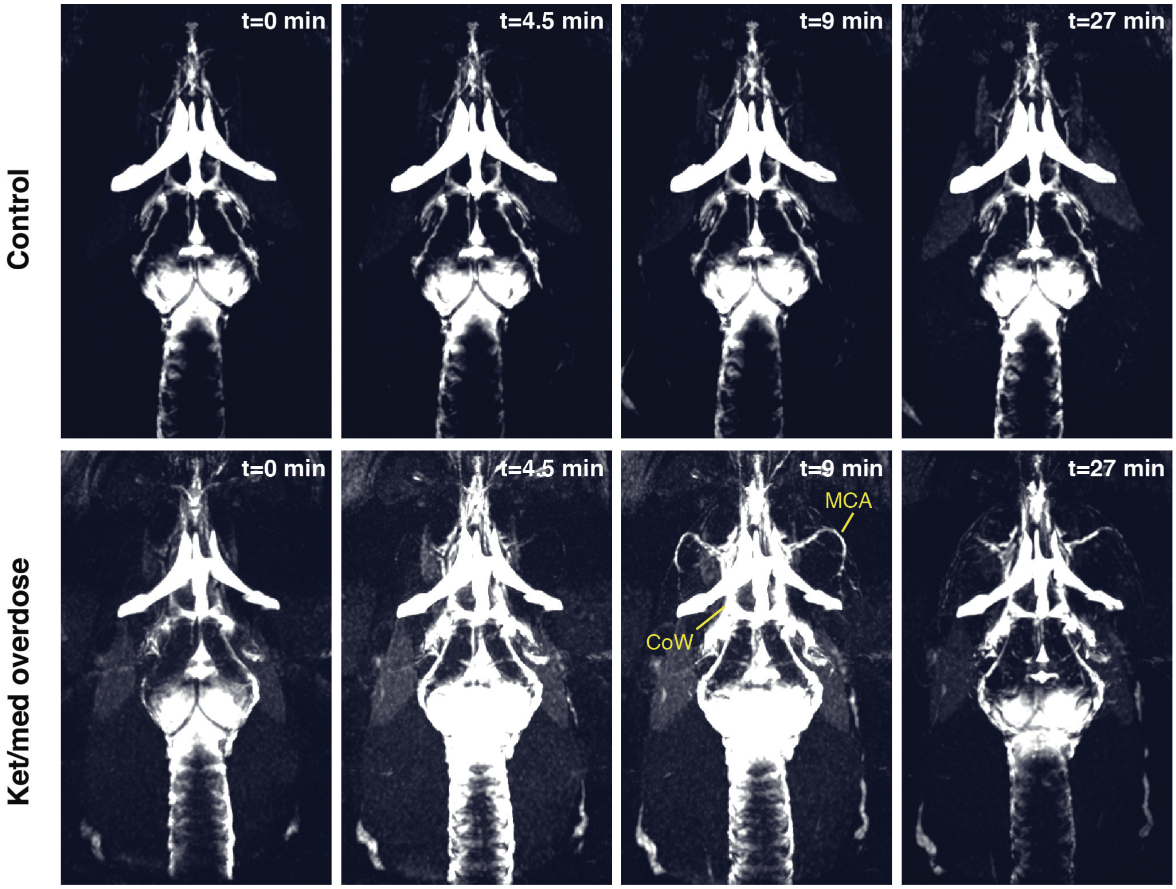

Fig. S3. Spread of contrast agent infused into the ventricles to PVS after death as detected with MRI

Visualization of tracer spread after intraventricular infusion of 2.5 µL of a Gadospin D solution at 25 mM gadolinium; data acquired with a series of T1-weighted MRI measurements. On the upper panel, representative MIP images of mouse kept alive under ket/med anesthesia (n=5) show no spread of the tracer at the circle of Willis or to the middle cerebral artery. On the bottom panel, representative MIP images of a mouse that was overdosed with ket/med (n=5). The last breath is indicated as t=0. Strong enhancements of signal are detectable 9 min after the last breath at the circle of Willis (CoW) and at the middle cerebral artery (MCA). Smaller branches of the MCA are clearly visible 27 min after the last breath.

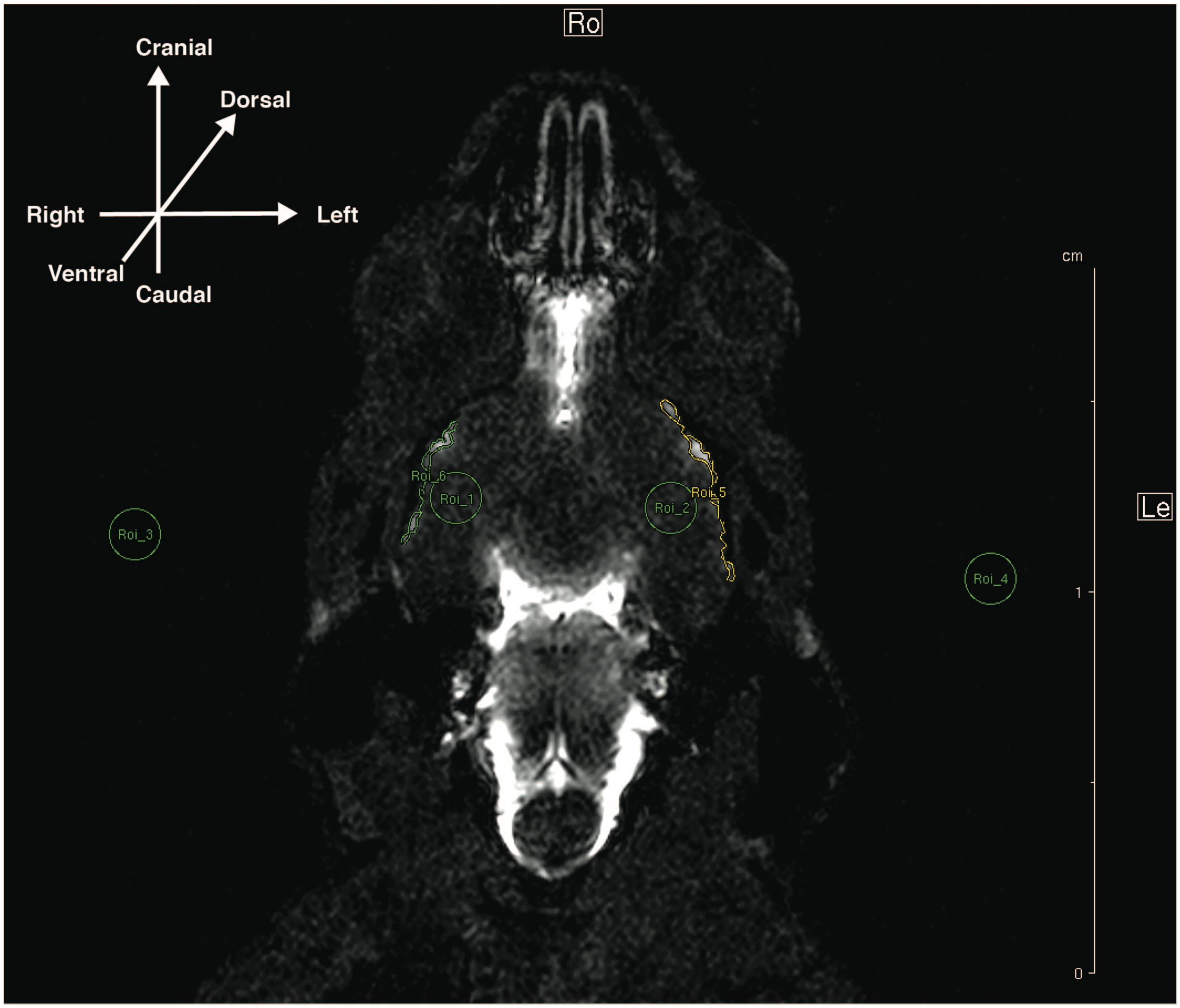

Fig. S4. Calculation of SNR from individual SI and noise measurements. For the animals injected in the cisterna magna with Gadospin D, SNR was calculated for PVS and brain parenchyma for both hemispheres. SI and noise measurements were obtained from MRI experiments performed at multiple timepoints, from single slices located 1 mm dorsal to the circle of Willis, by creating individual ROIs in the MR images (region of interest tool, image display and processing, ParaVision 6.0.1). Brain parenchyma mean SI was collected from circular ROIs placed close to the structures identified as cortical vessels (ROI_1 and ROI_2). Noise was measured as standard deviation (SD) of the background signal collected from two

ROIs placed independently outside the animals to the left and right (ROI 3, ROI 4). Circular ROIs for brain parenchyma SI and noise measurements were selected at identical sizes. PVS for cortical surface vessels was investigated in the territory of the middle cerebral artery. Target structures were identified close to the brain surface and presenting hyperintense when compared with adjacent brain tissue (ROI 5, ROI 6). Segmentation was performed manually. SNR was calculated for brain parenchyma and PVS for each time point investigated as follows:

$$SNR = ((\text{Mean } SI_{hemisphere\_1} / \text{Mean } SD_{hemisphere\_1}) + (\text{Mean } SI_{hemisphere\_2} / \text{Mean } SD_{hemisphere\_2})) / 2$$

SNR for the different timepoints were normalized to the time of death (set to t=0).

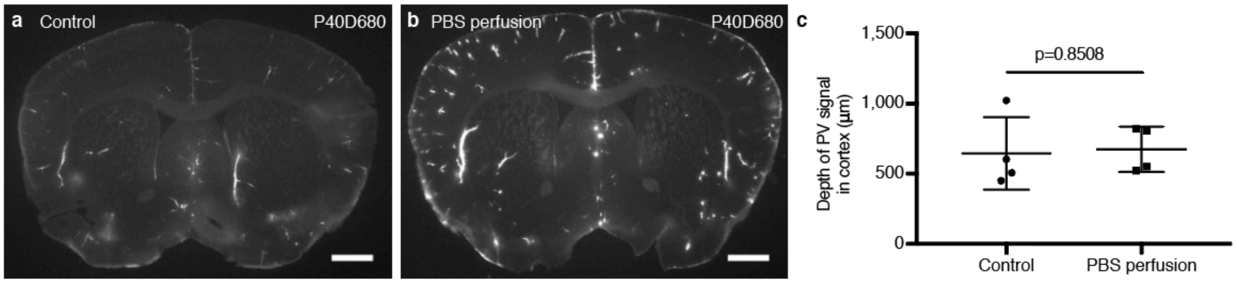

Fig. S5. Transcardiac perfusion does not alter depth of tracer penetration into the cerebral cortex. a-b, Representative images of 100 μm brain sections from mice sacrificed 60 min after i.c.v. infusion of 2.5 μL 200 μM P40D680 by overdose with ket/med (**a**) or overdose with ket/med followed by transcardiac perfusion with ice-cold PBS (**b**). Scale bars: 1000 μm. Note differences in image contrast that are due to lack of blood in vessels of perfused mouse. **c,** Quantification of depth of tracer penetration into the cerebral cortex.

Supplementary Video Legends

Video S1

Model of the relationship between CSF outflow and PVS spread. During awake conditions, tracer-filled CSF rapidly flows through the basal cisterns and effluxes along perineural routes to lymphatic vessels outside the skull. The rapid washout from the subarachnoid space leads to minimal spread of tracers to the PVS of the brain when assessed ex vivo. During anesthetized conditions, tracer-filled CSF is slower to efflux and is retained over time in the basal cisterns. This results in greater presence of tracers in the PVS when assessed ex vivo.

Video S2

Video showing spread of tracer along brain surface blood vessels after infusion of 2.5 μL 200 μM P40D800 into the contralateral ventricle. Time after end of infusion indicated. Scale bar: 1000 μm. Images are acquired at 1 frame per 15 s at 20x magnification.

Video S3

Video showing spread of tracer along both surface arteries and veins 60 min after i.c.v. infusion of 2.5 μL 200 μM P40D800. Dorsal middle cerebral vein, dorsal rostral cerebral vein and branches of the MCA showing tracer signal are indicated in the video. Images are acquired at 2.5 frames per s at variable magnification.

Video S4

Video showing pressure wave propagating along MCA branches and subsequent tracer spread to penetrating blood vessels following overdose with ket/med 60 min after infusion of 2.5 μL 200

μM P40D800 into the contralateral ventricle. Time relative to last breath indicated. Scale bar: 500 μm. Images are acquired at 1 frame per 2.5 s at 30x magnification.

Video S5

Representative video (n=5) showing spread of tracer after infusion of 5 μL of a Gadospin D solution at 25 mM gadolinium into the cisterna magna acquired with a sequence of T1-weighted MRI acquisitions. The mouse was kept alive under ket/med and showed no spread of the tracer towards the PVS. Images are acquired at 1 frame (color-coded MIP) per 4.5 min.

Video S6

Representative video (n=5) showing spread of tracer after infusion of 5 μL of a Gadospin D solution at 25 mM gadolinium into the cisterna magna acquired with a sequence of T1-weighted MRI acquisitions. The mouse was overdosed with ket/med and death is indicated as t=0. Strong enhancements of signal are detectable 9 min after death at the circle of Willis and at the middle cerebral artery. Smaller branches of the middle cerebral artery start to be visible 18 min after death. Images are acquired at 1 frame (color-coded MIP) per 4.5 min.

Video S7

Video showing continued spread of tracer to penetrating blood vessels after transcardiac perfusion with ice-cold PBS 60 min after infusion of 2.5 μL 200 μM P40D800 into the contralateral ventricle. Time relative to start of video indicated. Scale bar: 1000 μm. Images are acquired at 1 frame per 2.5 s at 20x magnification.
